# Toripalimab combined with FLOT chemotherapy as conversion therapy for gastric cancer with peritoneal metastasis: a single-arm, open-label, phase II trial

**DOI:** 10.1186/s12885-025-15166-w

**Published:** 2025-11-08

**Authors:** Zijing Zhang, Zeyu Lin, Yuting Xu, Lijie Luo, Haiping Zeng, Wenjun Xiong, Yan Chen, Yaohui Peng, Tingting Yang, YanSheng Zheng, Jin Li, Haipeng Huang, Wei Wang

**Affiliations:** 1 Department of Gastrointestinal Surgery, The First Affiliated Hospital of Guangzhou University of Chinese Medicine, Guangdong Clinical Research Academy of Chinese Medicine, Guangzhou, China; 2 The First Clinical Medical School of Guangzhou University of Chinese Medicine, Guangzhou, China; 3 Anorectal Department, Zhongshan Hospital of Chinese Medicine, Zhongshan, China; 4 Second Department of General Surgery, Zhongshan City People’s Hospital, Zhongshan, China; 5 Department of Gastrointestinal Surgery, The First Affiliated Hospital of Jinan University, Guangzhou, China; 6 Department of Colorectal Surgery, The Second Affiliated Hospital of Guangzhou University of Chinese Medicine, Guangdong Provincial Hospital of Chinese Medicine, Guangzhou, China

**Keywords:** Gastric cancer, Peritoneal metastasis, Conversion therapy, Toripalimab, FLOT

## Abstract

**Background:**

The combination of PD-1/PD-L1 monoclonal antibodies and chemotherapy has established a new standard of care for the first-line treatment of patients with unresectable locally advanced or metastatic gastric cancer (GC) and gastro-oesophageal junction adenocarcinoma. However, peritoneal metastasis represents a distinct pattern of dissemination in GC, typically associated with a poor prognosis. Whether the combination regimen improves survival for patients with concomitant peritoneal metastasis remains controversial. This study aims to evaluate the efficacy and safety of toripalimab (an anti-PD-1 monoclonal antibody) combined with FLOT chemotherapy as conversion therapy in these patients.

**Methods:**

In this single-arm, open-label, phase II trial conducted in China, we enrolled patients aged 18–80 years with laparoscopically proven gastric cancer and peritoneal metastasis. Patients received toripalimab (3 mg/kg) plus FLOT chemotherapy (docetaxel 50 mg/m^2^; oxaliplatin 85 mg/m^2^; leucovorin 200 mg/m^2^, 5-FU 2600 mg/m^2^) every 14 days for up to 4 cycles, followed by surgical resection. Patients who underwent surgery subsequently received 4 cycles of adjuvant treatment. The primary endpoint was the R0 resection conversion rate. The secondary endpoints included progression-free survival (PFS), overall survival (OS) and safety.

**Results:**

Between April 2021 and April 2023, 24 patients were screened, 20 of whom were included in this analysis. The median follow-up was 10.8 months. The objective response rate (ORR) was 35% and the disease control rate (DCR) was 80%. The R0 resection conversion rate after treatment was 25% (5/20), 40% (2/5) participants achieved TRG1 and 60% (3/5) participants achieved TRG2. The median PFS and OS were 6.5 and 10.8 months, respectively. Grade 3–4 treatment-related adverse events (TRAEs) occurred in 35% of participants.

**Conclusions:**

Toripalimab combined with FLOT chemotherapy demonstrated potential conversion efficacy in the treatment of gastric cancer with peritoneal metastasis.

**Clinical trial information:**

ClinicalTrials.gov (NCT04886193). Date of registration: 13 May 2021.

**Supplementary Information:**

The online version contains supplementary material available at 10.1186/s12885-025-15166-w.

## Introduction

Gastric cancer (GC), a common malignant tumor, continues to exhibit high global incidence and mortality rates [1, 2]. Peritoneal metastasis is a common form of metastasis or recurrence in GC, and is one of the leading causes of death in advanced GC patients. The prognosis for patients with GC and peritoneal metastasis is extremely poor, with most having a survival time of less than 1 year [3, 4].

With recent advances in systemic chemotherapy, conversion therapy plus surgery might be associated with prolonged survival of patient with stage IV GC [5–7]. In the AIO-FLOT3 Trial [8], an exploratory, phase II study, patients with limited metastatic GC (arm B) who received FLOT chemotherapy followed by surgery achieved longer survival (median OS 31.3 months vs. 15.9 months). In arm B, the response rate reached 60%, and 36 of 60 patients underwent surgery. However, arm B included few patients with peritoneal carcinomatosis, and the efficacy of this regimen in this specific patients remains controversial.

Immune checkpoint inhibitors have been incorporated into the treatment in advanced gastric or esophagogastric junction (GEJ) cancer recently. Programmed cell death protein (PD-1) inhibition has been demonstrated to partially suppress the peritoneal metastasis from GC by remodeling the cellular immune composition of peritoneal tumors [9]. Clinically, the CheckMate-649, a phase Ⅲ study, in advanced GC/GEJC patients, combining chemotherapy with Nivolumab as first-line treatment demonstrated favorable outcomes in OS and objective response rate (ORR) [10]. Furthermore, results of the phase Ⅲ RATIONALE 305 trial, comparing tislelizumab or placebo combined with chemotherapy showed a significantly improved OS and a higher ORR with the PD-1 inhibitor–containing regimen, suggesting a trend toward reducing the risk of death in patients with peritoneal metastasis [11]. Additionally, a phase Ib/II clinical trial showed that toripalimab plus XELOX chemotherapy achieved a favorable efficacy in ORR (66.7%) among advanced GC patients [12].

We conducted this phase II study to evaluate the conversion efficacy after treatment with a PD-1 antibody (toripalimab) combined with FLOT chemotherapy in stage IV GC patients with peritoneal metastasis.

## Method

### Patients

This study is a single-arm, open-label, phase II clinical trial conducted at The Second Affiliated Hospital of Guangzhou University of Chinese Medicine (Guangdong Provincial Hospital of Chinese Medicine). Patients were between 18 and 80 years old with histologically and laparoscopically confirmed gastric adenocarcinoma with peritoneal metastasis by peritoneal nodule or exfoliative cytology testing. Patients were required to have an Eastern Cooperative Oncology Group Performance Status (ECOG PS) of 0 or 1; and to be eligible for surgery as evaluated by American Society of Anesthesiologists Score (ASA) between I and Ⅲ. Exclusion criteria included pregnant or lactating women, suffered from severe mental illness, distant metastases in the liver, lungs, or brain, other malignancy within the past 5 years; allergies to any component of toripalimab, docetaxel, oxaliplatin, and fluorouracil; continuous systemic corticosteroid therapy within 1 month, complications of GC (bleeding, perforation, obstruction) requiring emergency surgery; unstable angina or myocardial infarction, cerebral infarction or cerebral hemorrhage within 6 months, or a lung function test FEV1 < 50% of the expected value. Additional exclusion criteria included: enrollment in other clinical trial (observational trials or follow-up periods of interventional trials were permitted); prior anti-PD-1 or anti-PD-L1 antibody therapy; receipt of any of the following treatments within 4 weeks: investigational drug or anti-cancer treatment, anti-tumor vaccines or live vaccines, or major surgery or trauma).

The study protocol was approved by the ethics committee of The Second Affiliated Hospital of Guangzhou University of Chinese Medicine (BF2021-065-01). The study was executed in accordance with the ethical standards set forth in the Declaration of Helsinki, Good Clinical Practice (GCP) guidelines and local regulatory requirements. Written informed consent was obtained from all participants prior to their inclusion in the study. The trial is registered on ClinicalTrials.gov (NCT04886193).

### Procedures

Patients received conversion therapy for 8 weeks including toripalimab 3 mg/kg administered intravenously (IV) over 30 min and FLOT chemotherapy (docetaxel 50 mg/m^2^ iv d1; oxaliplatin 85 mg/m^2^ iv d1; leucovorin 200 mg/m^2^ iv d1; 5-FU 2600 mg/m^2^ civ 24 h) administered once every 2 weeks for up to four cycles. After this four cycles of treatment, tumor response was assessed by thoraco-abdomino-pelvic computed tomography (CT) scan. Surgical feasibility was subsequently determined by a multidisciplinary team meeting at the participating center. The main criteria for surgery were as follows: (1) The primary tumor had regressed by more than 50%, (2) No moderate or significant ascites or peritoneal metastasis were seen on CT, (3) No other distant metastasis, (4) The patient’s physical condition was able to tolerate the surgery. If surgery was not feasible for a patient, the most suitable regimen was determined by this multidisciplinary team. The other patients underwent R0 surgical resection with D2 lymph nodes dissection. Diagnostic laparoscopy is the initial step in every surgical procedure. Any finding of peritoneal dissemination (even small nodules not seen on CT) would result in the termination of the planned radical resection. Patients who underwent surgery subsequently received 4 cycles of adjuvant treatment. After completing the 4 doses of treatment, these patients received oral S-1 twice daily (BSA < 1.25 m^2^: 40 mg, 1.25 m^2^ ≤ BSA ≤ 1.5 m^2^: 50 mg, BSA > 1.5 m^2^: 60 mg) on days 1–14, and toripalimab 240 mg administered IV over 30 min once every 3 weeks for 12 months. Patients were assessed before each treatment cycle for adverse events (AEs), ECOG PS and laboratory values. Dose modifications were performed based on toxic effects (NCI CTCAE, version 4.03).

### Assessments and outcomes

The primary outcome was the surgical conversion rate defined as the proportion of patients who underwent R0 surgical resection by multidisciplinary assessment after receiving 4 cycles of conversion therapy and tumor regression grade (TRG) using the American Joint Committee on Cancer (AJCC) TRG grading system. The TRG was evaluated by a pathologist at the Pathological Department of The Second Affiliated Hospital of Guangzhou University of Chinese Medicine. Secondary endpoints included progression-free survival (PFS) defined as the time from first dose of treatment to first documented progression or death from any cause, OS defined as the time from the first dose of treatment to death from any cause, and AEs during treatment. Radiographic imaging was carried out post-treatment for combination therapy, and evaluated by investigators per RECIST v1.1.

### Statistical analysis

This study investigated the efficacy of toripalimab combined with FLOT chemotherapy as conversion therapy for stage IV GC patients with peritoneal metastasis. The primary endpoint was the R0 resection conversion rate. Based on historical data indication an approximately 5% conversion rate with systemic chemotherapy alone in advanced GC with peritoneal metastasis, this study hypothesized that the experimental regimen would achieve a conversion rate of 22.5%. A sample size of 20 subjects was determined to provide 80% power at a 0.05 significance level using a two-sided, normal approximation method. The primary efficacy analyses were performed on the Full Analysis Set (FAS), including all assigned eligible patients who received the treatment. Safety analysis was conducted on participants who received at least one cycle of therapy. Survival curves were estimated using the Kaplan-Meier method. The 95% confidence intervals for the median survival times were calculated using the Brookmeyer-Crowley method. Statistical analysis was conducted using SPSS software version 26 or SAS 9.4.

## Result

### Baseline patient characteristics

**Fig. 1:**
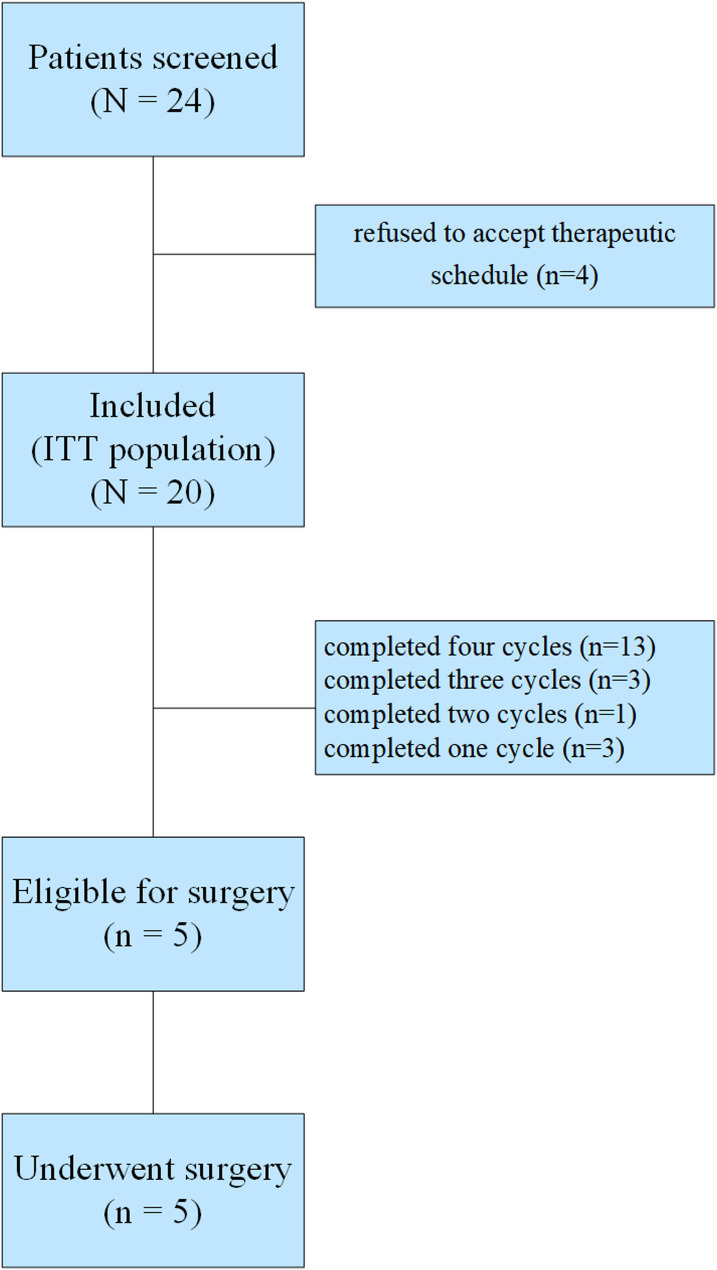
Flow chart

Between April 2021, and April 2023, 24 patients with stage IV GC with peritoneal metastasis were screened at The Second Affiliated Hospital of Guangzhou University of Chinese Medicine (Fig. 1). Of them, four refused to accept therapeutic schedule and were not included, leaving a total of 20 eligible GC patients with peritoneal metastasis who were enrolled and given the multimodality treatment schedule. At data cutoff (December 3, 2023), the median duration of follow-up (time from laparoscopy to the cutoff date) was 10.8 months (95% CI, 2.1 to 14.9). Demographic and baseline characteristics are listed in Table 1. All 20 patients underwent laparoscopy, which confirmed that 7 patients had a peritoneal carcinomatosis index (PCI) < 6, 8 patients had PCI ≥ 6 and < 10, and 5 patients had PCI ≥ 10. The ascitic volume of five patients was more than 1000 mL. Histological differentiation showed that 95.0% of tumors were poorly differentiated.

**Table 1:** Baseline characteristics

Characteristic	No. of Patients
Sex	
Male	10 (50)
Female	10 (50)
Median age, years (range)	54 (32–70)
BMI, mean ± SD (kg/m2)	20.96 ± 2.71
ECOG PS at baseline	
0	16 (80)
1	4 (20)
PCI	
PCI < 6	7 (35)
6 ≤ PCI < 10	8 (40)
PCI ≥ 10	5 (25)
Ascitic volume	
As < 1000 ml	15 (75)
As ≥ 1000 ml	5 (25)
Histologic differentiation*	
Differentiated	1 (5)
Undifferentiated	19 (95)

Data are No. (%) unless otherwise indicated

*ECOG PS* Eastern Cooperative Oncology Group Performance Status, *PCI* peritoneal carcinomatosis index, *As* Ascitic volume

*Differentiated, papillary adenocarcinoma and tubular adenocarcinoma (well differentiated, moderately differentiated); Undifferentiated, poorly differentiated adenocarcinoma (solid type, nonsolid type), signet ring cell carcinoma, and mucinous adenocarcinoma

### Conversion therapy

Of the included 20 patients, 13 (65%) completed the planned first four cycles of conversion therapy, three patient (15%) completed three cycles, one patient (5%) completed two cycles, and three patients (15%) completed one cycle. The overall incidence of AE was 95% (Table 2). Grade 3–4 TRAEs were recorded among seven patients (35%), leading to the discontinuation of conversion treatment in five patients before surgery. Treatment was discontinued in half of the cohort (ten patients) following conversion therapy. The most common grade 3–4 TRAEs during conversion therapy were neutropenia (*n* = 5). The RECIST 1.1 assessment was performed after conversion therapy for the intent-to-treat (ITT) population: partial response (*n* = 7), stable disease (*n* = 9), and progressive disease (*n* = 4), resulting in an ORR of 35.0% and a DCR of 80%.

**Table 2:** TRAEs during or 3 weeks after Toripalimab and FLOT chemotherapy or before surgery in the Intent-to-Treat population

TRAE	Any Grade, No.(%)	Grade 3–4, No.(%)
Any TRAE	19 (95)	7 (35)
TRAE Leading to Treatment Discontinuation	7 (35)	5 (25)
Leukopenia	19 (95)	4 (20)
Neutropenia	18 (90)	5 (25)
Fatigue	5 (25)	0 (0)
Vomiting	8 (40)	3 (15)
Diarrhea	6 (30)	1 (5)
Peripheral neuropathy	3 (15)	0 (0)
Alopecia	5 (25)	2 (10)

TRAEs were assessed during conversion therapy according to the National Cancer Institute Common Terminology Criteria for Adverse Events version 4.03. Categories are not mutually exclusive. Patients may be counted in more than one category for both Any TRAE and specific event types

*TRAE* treatment-related adverse event

### Surgery

After conversion therapy, a total of 5 patients (25%) underwent surgery determined by a multidisciplinary team meeting (Conversion group), while 15 (75%) did not (No-Conversion group). The detailed baseline characteristics stratified by groups are summarized in Table 3. Possibly due to the limited sample size, there were no statistically significant differences in baseline characteristics between the two groups. However, some trends were observed in the data: the conversion group tended to have lower PCI scores and less ascites. Total gastrectomy and D2 lymph node dissection were performed in all 5 patients and one patient also received para-aortic lymph node dissection (Table 4). No peritoneal metastasis was found in these patients. All these patients achieved negative cytology conversion. Two patients (40%) achieved TRG 1 and three patients (60%) achieved TRG 2. No Clavien–Dindo grade IIIa or higher postoperative complications were observed in any of the 5 patients.

**Table 3:** Baseline characteristics of patients who underwent surgery vs. Those who did not

Characteristic	Conversion group (*n* = 5)	No-Conversion group (*n* = 15)
Sex		
Male	2 (40)	8 (53.3)
Female	3 (60)	7 (46.7)
Median age, years (range)	56 (40–70)	49 (32–70)
BMI, mean ± SD (kg/m2)	21.00 ± 2.86	20.83 ± 2.53
ECOG PS at baseline		
0	4 (80)	12 (80)
1	1 (20)	3 (20)
PCI		
PCI < 6	1 (20)	6 (40)
6 ≤ PCI < 10	4 (80)	4 (26.7)
PCI ≥ 10	0	5 (33.3)
Ascitic volume		
As < 1000 ml	4 (80)	10 (66.7)
As ≥ 1000 ml	1 (20)	5 (33.3)
Histologic differentiation*		
Differentiated	0	1 (6.7)
Undifferentiated	5 (100)	14 (93.3)
conversion therapy		
completed	4 (80)	9 (60)
Incomplete	1 (20)	6 (40)

Data are No. (%) unless otherwise indicated

*ECOG PS* Eastern Cooperative Oncology Group Performance Status, *PCI* peritoneal carcinomatosis index, *As* Ascitic volume

*Differentiated, papillary adenocarcinoma and tubular adenocarcinoma (well differentiated, moderately differentiated); Undifferentiated, poorly differentiated adenocarcinoma (solid type, nonsolid type), signet ring cell carcinoma, and mucinous adenocarcinoma

**Table 4:** Pathologic characteristics of patients who underwent surgery

characteristic	Patients (*n* = 5), No. (%)
Total gastrectomy	5 (100)
Lymph nodes dissection	
D2	4 (80)
D2 + Para-aortic Lymph Node	1 (20)
ypT stage	
ypT0	1 (20)
ypT4	4 (80)
ypN stage	
ypN0	1 (20)
ypN1	1 (20)
ypN2	1 (20)
ypN3	2 (40)
TRG grade	
TRG 1	2 (40)
TRG 2	3 (60)

*TRG *grade tumor regression grade, *TRG 0* Complete regression without residual tumor cells, *TRG 1* Moderate regression with only single or small residual cancer cells visible, *TRG 2* Mild regression residual tumor but less than fibrotic stroma, *TRG 3* No regression extensive tumor residue no or minimal tumor cell necrosis

### OS and PFS

**Fig. 2:**
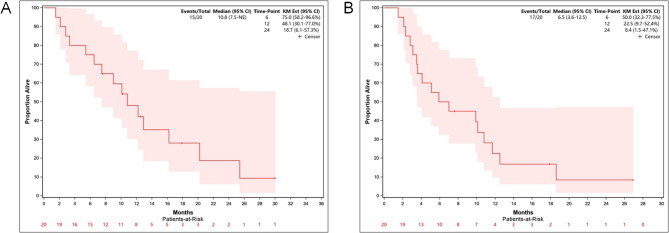
Clinical Outcomes of Patients Treated in the Trial. NOTE: The overall survival (OS) (**A**) and progression-free survival (PFS) (**B**) are presented for the participants

Twenty eligible patients were included the ITT population survival analysis (cutoff date: December 3, 2023). The Kaplan-Meier curves for OS and PFS are shown in Fig. 2. The participants achieved a median OS of 10.8 (95% CI, 7.5-NR) months with a 1-year survival of 48.1 (95% CI, 30.1–77.0%). The median PFS was 6.5 (95% CI, 3.6–12.5) months with a 1-year PFS of 22.5 (95% CI, 9.7–52.4%). Despite the limited sample size, which precludes definitive statistical conclusions, a trend toward improved survival was observed in patients who underwent conversion surgery (Supplementary Fig. 1). The curves show an early and sustained separation. The median OS was not reached in the conversion surgery group, with an estimated median OS of 25.4 months (95% CI, 20.2–NR), compared to 9.0 months (95% CI, 5.4–12.9) in the non-conversion group. Similarly, the median DFS was estimated to be 26.9 months (95% CI, 18.6–NR) in the conversion group, versus 4.1 months (95% CI, 3.1–10.8) in the non-conversion group. It is important to note that the median survival estimates and their associated confidence intervals in subgroup analyses should be interpreted with caution due to the limited number of events, resulting in wider confidence intervals and greater uncertainty in the point estimate.

## Discussion

This single-arm, open-label, phase II trial was conducted to evaluate the conversion efficacy of the combination of FLOT chemotherapy and toripalimab in the first-line treatment in GC patients with peritoneal metastasis. This regimen achieved a conversion rate of 25%. It resulted in a DCR of 80%, a 1-year survival rate of 48.1%, a median OS of 10.8 months, a 1-year PFS rate of 22.5% and a median PFS of 6.5 months. The main TRAEs were leukopenia and neutropenia. The overall incidence of Grade 3 or worse TRAEs was 35%. Patients who became resectable after conversion therapy showed a trend toward improved survival. To our knowledge, there are currently no prospective trials evaluating the conversion efficacy of the combination of FLOT chemotherapy and PD-1 inhibition in these patients.

Conversion therapy is aimed to achieving R0 resection after systemic therapy for tumors initially considered unresectable, which may lead to prolonged the survival of patients with peritoneal metastasis [13, 14]. It is considered an option for patients with advanced unresectable or recurrent GC who intend to achieve an R0 resection. A phase II single-arm clinical trial [15] showed a conversion rate of 18.4% (9/49) using intraperitoneal (IP) paclitaxel (PTX) plus SOX chemotherapy among GC patients with peritoneal metastasis. The DCR of this phase II trial was 76.6%, which is similar to the results of this study. Another prospective trial achieved a conversion rate of 36.7% (11/30) using the same regimen among GC patients with peritoneal metastasis; however, three patients also underwent ovary resection [16]. These studies showed similar conversion rate outcomes, indicating potential conversion efficacy in the treatment of GC with peritoneal metastasis. However, the results are still far from satisfactory, and their optimal application remains unclear. Further research is needed to explore selected populations and suitable regimens.

Although new medications have been developed in past decades, OS remains poor (median OS: range from 5.6 to 8.4 months) in GC patients with peritoneal metastasis receiving systemic chemotherapy compared with other metastatic sites [4, 17–19]. Compared with these results, our survival results were more encouraging with a longer median OS. A multicenter retrospective study of Nivolumab Plus mFOLFOX6 reported median PFS of 7.4 months and OS of 10.7 months, which are comparable to the results of this study [20]. This suggests that toripalimab added to chemotherapy might be a viable option for GC patients with peritoneal metastasis. In recent years, IP chemotherapy has gradually garnered attention among clinical physicians. Multiple phase II studies have been conducted in GC patients with peritoneal metastasis. The median PFS was similar to that reported in previous studies, but the median OS was slightly less than that reported in previous studies [15, 16]. Differences in patient selection and treatment rate may have contributed to these differences. The PCI score is a vital element that affects patients’ survival [21], and patients in the entire cohort with higher PCI scores may have contributed to the worse OS. In addition, the lower conversion therapy completion rate and second-line treatment rate may also contribute to this result. The conversion group demonstrated superior survival outcomes, which may be attributed to the lower PCI scores and ascites volume observed in these patients. Nevertheless, this regimen showed the potential for disease control and improving survival in GC patients with peritoneal metastasis.

This regimen combining toripalimab with systemic chemotherapy appeared safe. The overall incidence of Grade 3 or worse TRAEs was 35%, with the main TRAEs being leukopenia and neutropenia, which is consistent with findings from previous FLOT study [22]. However, 95% of patients experienced various degrees of complications and almost all participants experienced leukopenia and neutropenia. This is most likely correlated with the high tumor burden. Additionally, the combination of three chemotherapy drugs may have contributed to this poor tolerance among Asian patients. Furthermore, in these patients, emaciation (with a mean BMI of 20.96) due to growth of the primary lesions made it difficult to tolerate this treatment. Regarding the surgery, no Clavien–Dindo grade IIIa or higher postoperative complications were observed in all 5 patients, which demonstrated the safety of conversion surgery post-toripalimab plus FLOT chemotherapy.

Our study has some limitations. First, it was a single-arm phase II study, which meant its comparisons were with historical controls. Another limitation was the limited patient number for the exploratory phase II design and the enrollment of all patients from a single center. Additionally, The sample size was initially calculated using a two-sided normal approximation test, which may not be an appropriate method for small cohorts. A post-hoc assessment using simulation of the exact binomial test in R (10000 replicates) indicated that the achieved power was 69.0% to detect the hypothesized effect size. While below the conventional 80% threshold, this power was deemed acceptable given the exploratory nature of this phase II trial, which was aimed at gathering preliminary evidence, and the significant challenges in recruiting this specific patient population, advanced gastric cancer with peritoneal metastasis. Moreover, the limited sample size of patients in this study may impede the robustness of subgroup and multivariate analyses, thereby compromising the identification of independent predictors. As routine second-look laparoscopy was not required prior to cytoreductive surgery, patients with occult unresectable disease may have been overlooked. To minimise this risk, we adopted diagnostic laparoscopy as the initial step in our surgical process. Although this approach effectively prevented surgeries with minimal therapeutic benefit, it inevitably introduced selection bias. The positive outcomes reported in this study should be interpreted within this specific context and are primarily applicable to the selected patient population. Looking ahead, we advocate incorporating second-look laparoscopy in the design of future prospective trials, as this would enhance the quality of evidence obtained.

## Conclusion

To our knowledge, this trial is the first prospective study to evaluate toripalimab plus chemotherapy followed by surgery in GC patients with peritoneal metastasis. The results reported herein showed potential for conversion efficacy and improved survival in the treatment of GC with peritoneal metastasis, while maintaining a manageable safety profile. Further clinical trials, particularly those involving a larger cohort of participants, are needed to confirm our results before this regimen can be considered as the standard of care.

## Supplementary Information

Supplementary Material 1.

## Data Availability

The data that support the findings of this study are available from the corresponding author upon request.

## Abbreviations

As: Ascitic volume
ASA: American Society of Anesthesiologists Score
CT: Computed tomography
DCR: Disease control rate
ECOG PS: Eastern Cooperative Oncology Group Performance Status
GC: Gastric cancer
GEJC: Esophagogastric junction cancer
intent-to-treat: ITT
IV: Administered intravenously
ORR: Objective response rate
OS: Overall survival
PCI: Peritoneal carcinomatosis index
PD-1: Programmed cell death protein
PFS: Progression-free survival
TRAEs: Treatment-related adverse events
TRG grade: Tumor regression grade

## References

[1] Sung H, Ferlay J, Siegel RL, Laversanne M, Soerjomataram I, Jemal A, et al. Global cancer statistics 2020: GLOBOCAN estimates of incidence and mortality worldwide for 36 cancers in 185 countries. CA Cancer J Clin. 2021;71(3):209–49. 33538338 10.3322/caac.21660

[2] Zheng R, Zhang S, Zeng H, Wang S, Sun K, Chen R, et al. Cancer incidence and mortality in China, 2016. J Natl Cancer Cent. 2022;2(1):1–9. 39035212 10.1016/j.jncc.2022.02.002 PMC11256658

[3] Ajani JA, Rodriguez W, Bodoky G, Moiseyenko V, Lichinitser M, Gorbunova V, et al. Multicenter phase III comparison of cisplatin/S-1 with cisplatin/infusional fluorouracil in advanced gastric or gastroesophageal adenocarcinoma study: the FLAGS trial. J Clin Oncol. 2010;28(9):1547–53. 20159816 10.1200/JCO.2009.25.4706

[4] Koizumi W, Narahara H, Hara T, Takagane A, Akiya T, Takagi M, et al. S-1 plus cisplatin versus S-1 alone for first-line treatment of advanced gastric cancer (SPIRITS trial): a phase III trial. Lancet Oncol. 2008;9(3):215–21. 18282805 10.1016/S1470-2045(08)70035-4

[5] Yamaguchi K, Yoshida K, Tanahashi T, Takahashi T, Matsuhashi N, Tanaka Y, et al. The long-term survival of stage IV gastric cancer patients with conversion therapy. Gastric Cancer. 2018;21(2):315–23. 28616743 10.1007/s10120-017-0738-1 PMC5846815

[6] Sun J, Song Y, Wang Z, Chen X, Gao P, Xu Y, et al. Clinical significance of palliative gastrectomy on the survival of patients with incurable advanced gastric cancer: a systematic review and meta-analysis. BMC Cancer. 2013;13:577. 24304886 10.1186/1471-2407-13-577 PMC4235220

[7] Sato Y, Ohnuma H, Nobuoka T, Hirakawa M, Sagawa T, Fujikawa K, et al. Conversion therapy for inoperable advanced gastric cancer patients by docetaxel, cisplatin, and S-1 (DCS) chemotherapy: a multi-institutional retrospective study. Gastric Cancer. 2017;20(3):517–26. 27553665 10.1007/s10120-016-0633-1

[8] Al-Batran SE, Homann N, Pauligk C, Illerhaus G, Martens UM, Stoehlmacher J, et al. Effect of neoadjuvant chemotherapy followed by surgical resection on survival in patients with limited metastatic gastric or gastroesophageal junction cancer: the AIO-FLOT3 trial. JAMA Oncol. 2017;3(9):1237–44. 28448662 10.1001/jamaoncol.2017.0515 PMC5824287

[9] Kumagai Y, Futoh Y, Miyato H, Ohzawa H, Yamaguchi H, Saito S, et al. Effect of systemic or intraperitoneal administration of Anti-PD-1 antibody for peritoneal metastases from gastric cancer. Vivo. 2022;36(3):1126–35. 10.21873/invivo.12811 PMC9087086 35478147

[10] Janjigian YY, Shitara K, Moehler M, Garrido M, Salman P, Shen L, et al. First-line nivolumab plus chemotherapy versus chemotherapy alone for advanced gastric, gastro-oesophageal junction, and oesophageal adenocarcinoma (CheckMate 649): a randomised, open-label, phase 3 trial. Lancet. 2021;398(10294):27–40. 34102137 10.1016/S0140-6736(21)00797-2 PMC8436782

[11] Rationale 305. Phase 3 study of Tislelizumab plus chemotherapy vs placebo plus chemotherapy as first-line treatment (1L) of advanced gastric or gastroesophageal junction adenocarcinoma (GC/GEJC). J Clin Oncol 41:286–286.

[12] Wang F, Wei XL, Wang FH, Xu N, Shen L, Dai GH, et al. Safety, efficacy and tumor mutational burden as a biomarker of overall survival benefit in chemo-refractory gastric cancer treated with toripalimab, a PD-1 antibody in phase Ib/II clinical trial NCT02915432. Ann Oncol. 2019;30(9):1479–86. 31236579 10.1093/annonc/mdz197 PMC6771223

[13] Wang Z, Chen JQ, Liu JL, Tian L. Issues on peritoneal metastasis of gastric cancer: an update. World J Surg Oncol. 2019;17(1):215. 31829265 10.1186/s12957-019-1761-y PMC6907197

[14] Yu P, Ding G, Huang X, Wang C, Fang J, Huang L et al. Genomic and immune microenvironment features influencing chemoimmunotherapy response in gastric cancer with peritoneal metastasis: a retrospective cohort study. Int J Surg. 2024 Jun 1;110(6):3504-3517. 10.1097/JS9.0000000000001281 10.1097/JS9.0000000000001281 PMC11175815 38502852

[15] Tu L, Zhang W, Ni L, Xu Z, Yang K, Gou H, et al. Study of SOX combined with intraperitoneal high-dose Paclitaxel in gastric cancer with synchronous peritoneal metastasis: A phase II single-arm clinical trial. Cancer Med. 2023;12(4):4161–9. 36161282 10.1002/cam4.5277 PMC9972103

[16] Shi M, Yang Z, Lu S, Liu W, Ni Z, Yao X, et al. Oxaliplatin plus S-1 with intraperitoneal Paclitaxel for the treatment of Chinese advanced gastric cancer with peritoneal metastases. BMC Cancer. 2021;21(1):1344. 34922478 10.1186/s12885-021-09027-5 PMC8684127

[17] Sadeghi B, Arvieux C, Glehen O, Beaujard AC, Rivoire M, Baulieux J, et al. Peritoneal carcinomatosis from non-gynecologic malignancies: results of the EVOCAPE 1 multicentric prospective study. Cancer. 2000;88(2):358–63. 10640968 10.1002/(sici)1097-0142(20000115)88:2<358::aid-cncr16>3.0.co;2-o

[18] Thomassen I, van Gestel YR, van Ramshorst B, Luyer MD, Bosscha K, Nienhuijs SW, et al. Peritoneal carcinomatosis of gastric origin: a population-based study on incidence, survival and risk factors. Int J Cancer. 2014;134(3):622–8. 23832847 10.1002/ijc.28373

[19] Chau I, Norman AR, Cunningham D, Waters JS, Oates J, Ross PJ. Multivariate prognostic factor analysis in locally advanced and metastatic esophago-gastric cancer–pooled analysis from three multicenter, randomized, controlled trials using individual patient data. J Clin Oncol. 2004;22(12):2395–403. 15197201 10.1200/JCO.2004.08.154

[20] Nakayama Y, Ando T, Takahashi N, Tsukada K, Takagi H, Goto Y, et al. The efficacy and safety of nivolumab plus mFOLFOX6 in gastric cancer with severe peritoneal metastasis. J Clin Med. 2024;13(3):834. 38337528 10.3390/jcm13030834 PMC10856034

[21] Yang Z, Lu S, Shi M, Yuan H, Wang Z, Ni Z, et al. Oncological outcomes of conversion therapy in gastric cancer patients with peritoneal metastasis: a large-scale retrospective cohort study. Gastric Cancer. 2024;27(2):387–99. 38143257 10.1007/s10120-023-01452-8 PMC10896904

[22] Al-Batran SE, Hartmann JT, Hofheinz R, Homann N, Rethwisch V, Probst S, et al. Biweekly fluorouracil, leucovorin, oxaliplatin, and docetaxel (FLOT) for patients with metastatic adenocarcinoma of the stomach or esophagogastric junction: a phase II trial of the arbeitsgemeinschaft internistische onkologie. Ann Oncol. 2008;19(11):1882–7. 18669868 10.1093/annonc/mdn403

