# Supplementary material for: Toripalimab combined with FLOT chemotherapy as conversion therapy for gastric cancer with peritoneal metastasis: a single-arm, open-label, phase II trial
Source: BMC Cancer. 2025 Nov 8;25:1733. doi: 10.1186/s12885-025-15166-w (PMC12595676; doi:10.1186/s12885-025-15166-w)
Supplement: Supplementary file 1 — Supplementary Material 1. [file 12885_2025_15166_MOESM1_ESM.docx]

Supplementary figure 1. Survival Outcomes of Patients between the conversion and no-conversion groups


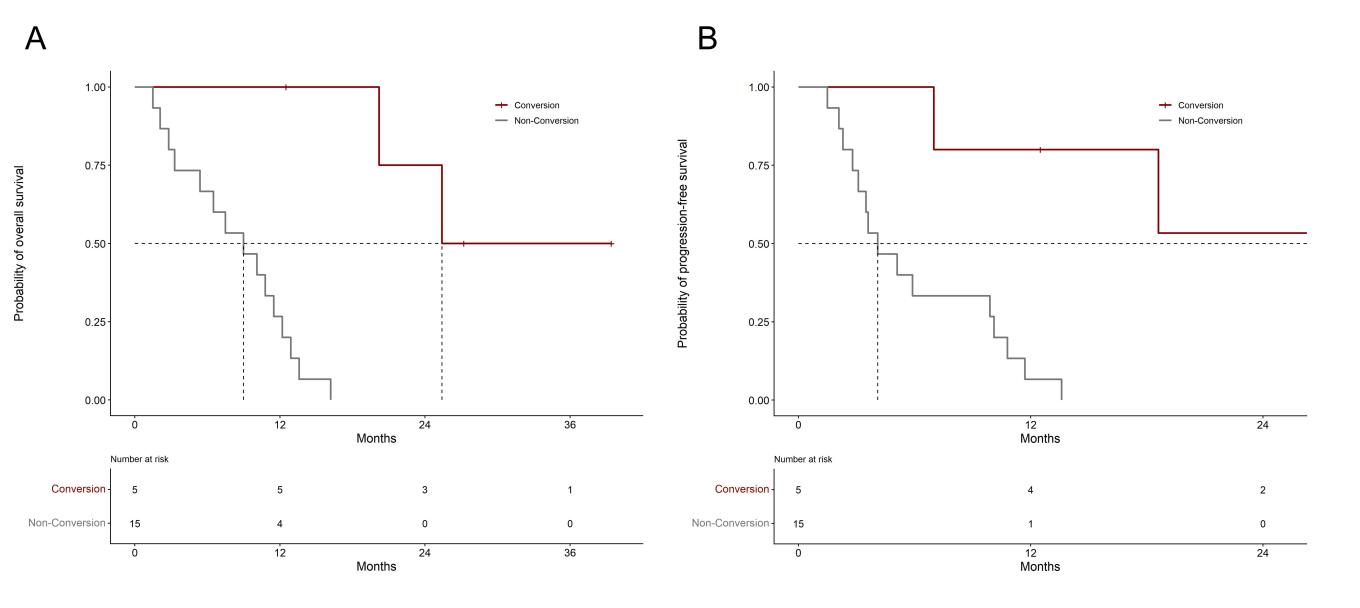


NOTES. The overall survival (OS) (A) and progression-free survival (PFS) (B) are presented for the participants. Due to small sample size (n=20), no formal statistical comparison was performed. Curves are presented for illustrative purposes only.
